# The ventral striatum contributes to the activity of the motor cortex and motor outputs in monkeys

**DOI:** 10.3389/fnsys.2022.979272

**Published:** 2022-08-29

**Authors:** Michiaki Suzuki, Yukio Nishimura

**Affiliations:** ^1^Division of Behavioral Development, Department of Developmental Physiology, National Institute for Physiological Sciences, Okazaki, Japan; ^2^Department of Physiological Sciences, School of Life Science, SOKENDAI, Hayama, Japan; ^3^Department of Neuroscience, Graduate School of Medicine, Kyoto University, Kyoto, Japan; ^4^Japan Society for the Promotion of Science, Tokyo, Japan; ^5^Neural Prosthetics Project, Department of Brain and Neurosciences, Tokyo Metropolitan Institute of Medical Science, Tokyo, Japan

**Keywords:** motor control, electrocorticogram (ECoG), non-human primate, effort, basal gangalia

## Abstract

The ventral striatum (VSt) is thought to be involved in the vigor of motivated behavior and is suggested to be a limbic-motor interface between limbic areas involved in motivational processes and neural circuits regulating behavioral outputs. However, there is little direct evidence demonstrating the involvement of the VSt in motor control for motivated behaviors. To clarify the functional role of the VSt in motor control, we investigated the effect of reversible pharmacological inactivation of the VSt on the oscillatory activity of the sensorimotor cortices and motor outputs in two macaque monkeys. VSt inactivation reduced movement-related activities of the primary motor cortex and premotor area at 15–120 Hz and increased those at 5–7 Hz. These changes were accompanied by reduced torque outputs but had no effect on the correct performance rate. The present study provides direct evidence that the VSt regulates activities of the motor cortices and motor output.

## Introduction

The behavioral functions of the ventral striatum (VSt) have been widely investigated in the field of neuroscience dealing with motivational processes for goal-directed behavior. The VSt is thought to be a “limbic-motor interface” between limbic areas involved in emotion and cognition and neural circuits regulating behavioral output (Mogenson et al., 1980). However, while the findings that the VSt facilitates the approach to reward-related stimuli are well documented (Aberman and Salamone, 1999; Salamone et al., 2001; Ishiwari et al., 2004; Ambroggi et al., 2008, 2011; Blaiss and Janak, 2009; Nicola, 2010; Khamassi and Humphries, 2012; Saunders and Robinson, 2012; Mannella et al., 2013; McGinty et al., 2013), there is little evidence demonstrating its role in motor function. It has been reported that VSt lesioning, inactivation, or the depletion of dopamine in this nucleus does not disrupt basic motor functions, such as feeding behavior and lever pressing, in intact animals (Aberman and Salamone, 1999; Salamone et al., 2001; Ishiwari et al., 2004; Sawada et al., 2015; Suzuki et al., 2020). Therefore, the VSt is not thought to be a part of a neural circuit regulating motor output (Floresco, 2015). Numerous rodent studies investigating the function of the VSt in goal-directed behaviors observed locomotor activity or simple response behavioral tasks, such as lever pressing, and did not measure any other information related to motor control (Aberman and Salamone, 1999; Salamone et al., 2001; Ambroggi et al., 2008, 2011; Blaiss and Janak, 2009; Nicola, 2010; Saunders and Robinson, 2012; McGinty et al., 2013). In such simple tasks, the functional contribution of the VSt in motor control might be masked. More detailed measurements of behavioral outcomes in the tasks are necessary to clarify whether the VSt contributes to motor control. In the present study, we trained macaque monkeys to perform a behavioral task requiring precise torque control of the forearm. Then, in order to clarify the functional role of the VSt in motor control, we recorded behavioral outcomes and brain activities from the sensorimotor cortices (SMC) before and during reversible pharmacological inactivation of the VSt.

## Materials and Method

### Animal preparation

Data were collected from two Japanese macaque monkeys [*Macaca fuscata*: Monk-1 (female, 5.0 kg, 5 years old) and Monk-2 (male, 6.8 kg, 6 years old)] provided through the National BioResource Project of the Japan Ministry of Education, Culture, Sports, Science and Technology (MEXT). All animal experimental procedures were approved by the Committees for Animal Experiments at the National Institute of Natural Sciences and at the Graduate School of Medicine in Kyoto University and were performed in accordance with the National Institutes of Health Guide for the Care and Use of Laboratory Animals. The animals were fed regularly with diet pellets. Monk-1 was under water-restriction for the behavioral task, while Monk-2 had free access to water. The monkeys were prepared using standard surgical techniques described elsewhere (Sawada et al., 2015). Briefly, a head-holder, a chamber located over the VSt to gain easy access for muscimol injections, and an electrocorticographic (ECoG) electrode array comprised of 12 channel (4 × 3 grid) electrodes over the forelimb area of the primary motor cortex (M1), the primary somatosensory cortex (S1), and premotor cortex (PM) on the left hemisphere, were implanted under isoflurane anesthesia and aseptic conditions.

### Behavioral task

**Figure 1:**
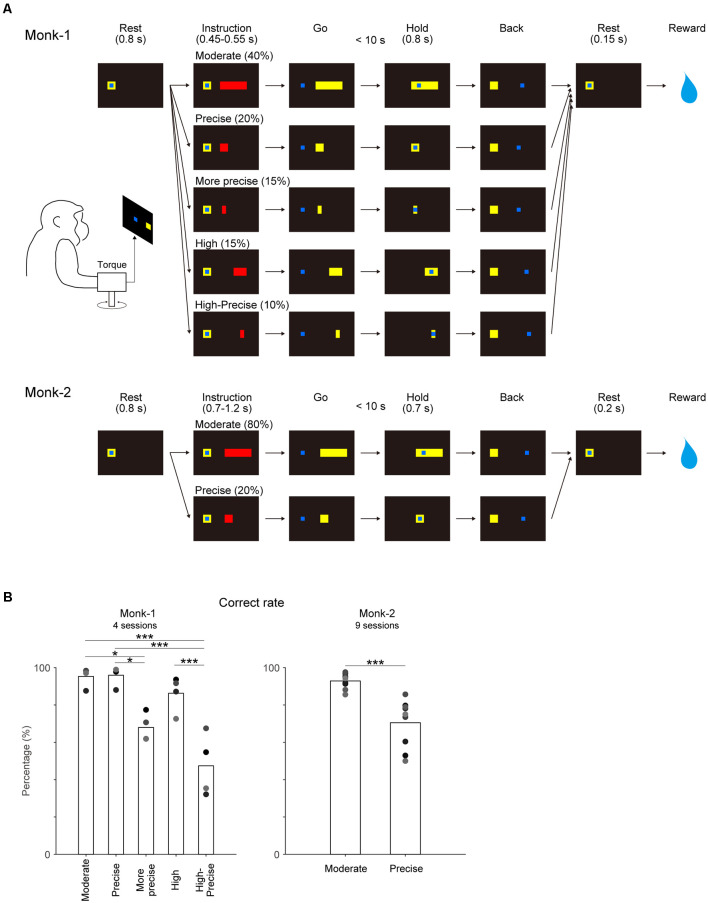
Behavioral task and its performance. **(A)** Each monkey controlled the position of the cursor (blue square) using wrist torque to acquire targets (yellow) displayed on the screen. After target instruction (red) for a preset time, the target color changed from red to yellow (go cue). Moderate target accepted the wide range of torque output by auxotonic torque production (Monk-1) or isometric torque production (Monk-2) about the wrist joint. Precise target required precise control of torque output. Additional three targets were imposed for Monk-1 as follows: More precise target required more precise control, High target required high torque output, and High-Precise target required precise control of high torque output. The probability of each target appearance is shown. **(B)** Correct rate for each target. Individual gray dots indicate the results of each session. One-way factorial ANOVA and *post hoc* comparison with Bonferroni correction for Monk-1, and paired *t*-test for Monk-2 were conducted. *P < 0.05, ***P < 0.001.

The manipulandum, which can measure wrist joint torque and joint angle in two dimensions (x, flexion-extension; y, ulnar-radial direction), was used for the present study. The monkeys controlled the one-dimensional (flexion-extension) position of a blue cursor on a video monitor with auxotonic (spring-loaded) contraction (Monk-1) or with isometric contraction of the wrist joint (Monk-2) and acquired targets displayed on the screen (Figure 1A). The cursor was adjusted to the rest position when the wrist joint torque was neutral. When the cursor stayed on the rest position for 0.8 s, one of the red targets appeared as an instruction cue. After an additional stay in the rest position for 0.45–0.55 s (Monk-1) or 0.7–1.2 s (Monk-2) during the instruction period, the target color changed from red to yellow, indicating a go-cue. Then, the monkeys were required to move and maintain the cursor on the target for 0.8 s (Monk-1) or 0.7 s (Monk-2) within 10 s after the go cue. After the acquisition of the target, the monkeys were required to back the cursor to the rest position and maintain this position for 0.15–0.2 s to receive a liquid (Monk-1) or apple sauce reward (Monk-2). Monk-1 and Monk-2 performed a five-graded and a two-graded torque-tracking task, respectively. The Moderate target accepted a wide range of torque output by auxotonic torque production (Monk-1) or isometric torque production (Monk-2) about the wrist joint. The Precise target required precise control of torque output. Since motor control of auxotonic contraction is assumed to be easier compared with isometric contraction, an additional three targets were imposed for Monk-1, as follows. A More precise target requiring more precise control, a High target requiring high torque output, and a High-Precise target requiring precise control of high torque output. Each of the targets was presented in randomized order with a preset probability. Monk-1 and Monk-2 performed four and nine sessions in the control conditions and five and five sessions in the VSt inactivation sessions, respectively (Supplementary Table 1). Monk-2 also performed one saline injection session.

### VSt inactivation

**Figure 2:**
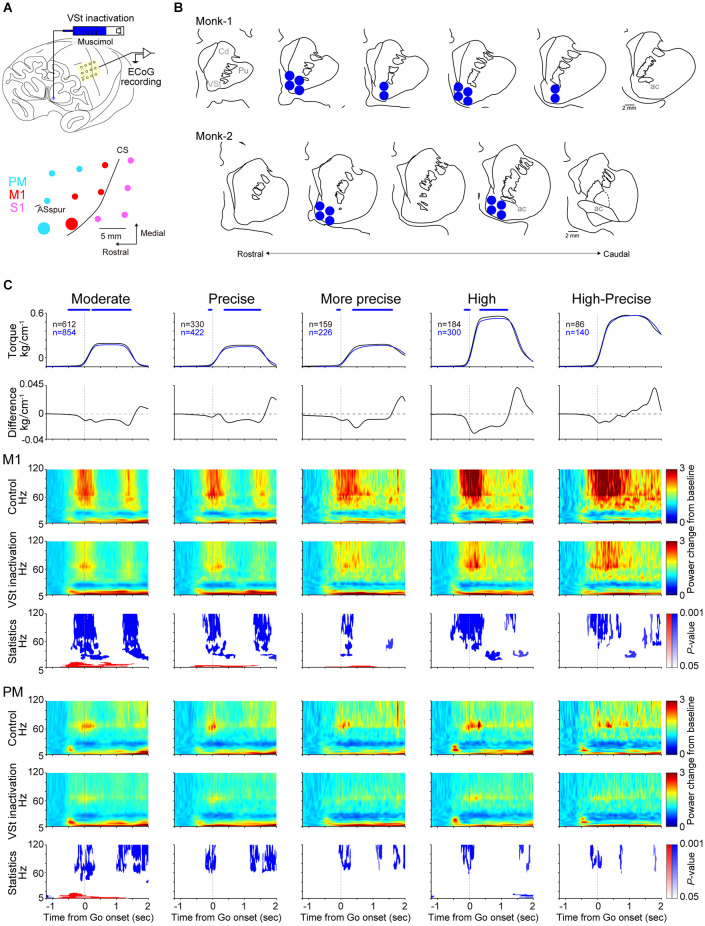
VSt inactivation reduces torque output and movement-related activity. **(A)** Top: Scheme of VSt inactivation and simultaneous ECoG recording. Bottom: Recording sites on the PM, M1, and S1 are shown in cyan, red, and pink color, respectively. The electrode locations on the functional map were determined relative to anatomical landmarks such as the central sulcus and arcuate sulcus and the location of the electrode. The activity of typical examples in **(C)** was recorded from the enlarged red (M1) and cyan (PM) sites. Data obtained from Monk-1. CS, central sulcus; ASspur, spur of the arcuate sulcus. **(B)** Muscimol injection sites reconstructed from histological coronal sections in both monkeys. Each section interval is 1 mm. Cd, Caudate nucleus; Pu, Putamen. **(C)** Comparison of movement-related activity of a typical example in the M1 and PM between control and the VSt inactivation trials. First row: Averaged wrist torque trace (Control, black; VSt inactivation, blue). Statistical difference between control and inactivation was investigated by Wilcoxon-signed rank test for wrist torque values from 0.5 s before to 2 s after go cue (627 time points) were conducted with Bonferroni correction (Control vs. VSt inactivation). Blue asterisks indicate a significant reduction in torque output during the VSt inactivation (*P* < 0.001). There were no time points with higher torque output during VSt inactivation compared with the control trials. Second row: Difference of torque traces between the inactivation and control (VSt inactivation minus Control). A negative value indicates reduced torque output during the VSt inactivation. Third, fourth, sixth, and seventh rows: Averaged movement-related power change of oscillatory cortical activity during the control (M1, third; PM, sixth row) and VSt inactivation (M1, fourth; PM, seventh row) trials. Each time-frequency representation value was divided by the mean baseline (rest period: −1.2 s to −0.6 s before go cue) value, then averaged across trials (see also “ECoG analysis” in “Materials and methods” Section). Fifth and eighth rows: Statistical *P*-value representation in time-frequency domain (M1, fifth; PM, eighth row). Blue and red areas indicate statistically inactivated and activated components during the VSt inactivation, respectively. Trials showing abnormal spectra were excluded using an automated algorithm from the EEGLAB library, pooled across sessions, and analyzed.

To investigate the contribution of the VSt to motor control, the VSt in the left hemisphere, contralateral to the task-engaged right forelimb, was inactivated pharmacologically. Muscimol, a gamma aminobutyric acid (GABA)_A_ receptor agonist [5 μg/μl, dissolved in 0.1 M phosphate buffer (PB, pH 7.4)], was pressure injected at a rate of 0.4 μl/min (2–8 sites, 0.5–2 μl/site, 2–4 μl/session) to focally inactivate the contralateral VSt (Figures 2A,B). Considering that a 1.5 μl injection of muscimol inactivates a 3–4 mm in diameter spherical volume of brain tissue (Murata et al., 2015), which is consistent with those of other previous studies (Martin, 1991; Arikan et al., 2002), injection sites and muscimol volumes (2–8 sites, 0.5–2 μl/site, 2–4 μl/session) were determined to infiltrate the entire VSt with muscimol, assuming that 1 μl of muscimol would diffuse within a 2 mm radius. A 10 μl Hamilton syringe (Hamilton Company, Reno, Nevada, USA) was held on a syringe pump fixed on the stereotaxic manipulator. The grid-guided needle was inserted stereotaxically into the VSt. Injection sites were estimated by MRI images. As a vehicle condition, saline was injected in Monk-2. The behavioral session began approximately 10 min after the infusion was complete.

### Data collection

During the experiments, task parameters such as target positions, timing of trial events, wrist torque (flexion-extension direction), and ECoG data were recorded simultaneously using a Cerebus multichannel data acquisition system (Blackrock Microsystems, Salt Lake City, UT, USA) at a sampling rate of 2 kHz.

### Data analysis

To compare behavioral outcomes between control (no intervention and/or vehicle) and the VSt inactivation sessions, we analyzed the initial 30-min data from each session.

#### Behavioral analysis

Correct rate was calculated as the number of correct trials divided by the total number of trials. Peak torque was defined as the maximum torque value (kg/cm^−1^) during the hold phase on the target. Reaction time (RT) was defined as the time window between the timing of the go cue and the timing when the cursor exceeded the right side of the rest position.

#### Statistics for behavioral outcome

To compare the correct rate for each target in control sessions, one-way factorial analysis of variance (ANOVA) and *post hoc* multiple comparisons with Bonferroni correction for Monk-1 and paired *t*-test for Monk-2 were conducted. To determine the effect of the VSt inactivation on correct rate, peak torque, and RT, a nonparametric Wilcoxon signed-rank test was conducted with Bonferroni correction (control vs. VSt inactivation for each target).

#### ECoG analysis

The data were downsampled eight times, resulting in a sampling rate of 250 Hz. Channels and trials with abnormal spectra were rejected using an automated algorithm from the EEGLAB library (Delorme et al., 2011). The ECoG signals were then aligned using the timing of instruction and go cues, respectively. Further analysis of cortical activity was performed using the Matlab FieldTrip toolbox (Oostenveld et al., 2011). The dynamics of cortical activity were quantified by the time-frequency representation (TFR) generated by Morlet wavelet transformation at 116 different center frequencies (5–120 Hz). Each TFR represented the in-trial dynamics, and they were pooled across sessions. For population analysis across recording areas, we defined five frequency bands as follow: θ (5–7 Hz), α (8–14 Hz), β (15–30 Hz), γ (31–70 Hz), and high-γ (71–120 Hz). In the case of Monk-2, data at 55–80 Hz were removed because of extraordinal signal contamination. To quantify movement-related power changes, we further divided each TFR value by the mean TFR value at the corresponding frequency during the rest period (baseline, −0.7 to −0.1 s before the instruction cue). To compare the movement-related power change in each band between control and the VSt inactivation trials, the movement-related power change from −0.5 to 0.5 s (Monk-1) or 0–1 s (Monk-2) around the go cue were averaged. Furthermore, to investigate the impact of VSt inactivation on brain activity in the rest period, TFR values during the rest period (−0.7 to −0.1 s before the instruction cue) in each frequency band were compared between the control and VSt inactivation trials.

#### Statistics for brain activity

Nonparametric Wilcoxon-signed rank test for each frequency domain was conducted to compare the movement-related power changes and TFR values during the rest period between the control and VSt inactivation trials. Furthermore, to compare movement-related activity in the time window before and after the go cue (−1.2 to 2 s from the go cue) between the control and VSt inactivation trials, the cluster-based permutation test was performed on movement-related activity obtained from the representative channels on the M1 and premotor area (PM) using the Matlab FieldTrip toolbox (Oostenveld et al., 2011). The number of permutations was set at 1,000 times. The *p*-value for cluster level and for permutation test was set at 0.05.

### Histology

To confirm the muscimol injection sites, histological analysis was conducted. Detailed procedures are described elsewhere (Sawada et al., 2015). Briefly, at the end of the experiment, electrocoagulation for the identification of muscimol injection sites in the VSt was made using a rectangular constant current at 40 μA for 20 s through the stimulating electrode under deep anesthesia with an overdose of sodium pentobarbital (50 mg/kg, i.v). Then, the monkeys were euthanized by transcardial perfusion with 0.1 M phosphate-buffered saline (pH 7.4), followed by 4% paraformaldehyde in 0.1 M phosphate-buffer (PB, pH 7.4). The perfused brain was postfixed in the same fresh fixative overnight at 4°C, and saturated with 10% sucrose in 0.1 M PB, followed by saturation in 20% and 30% sucrose solutions. The perfused brain was cut serially into 50 μm coronal sections, and every fifth section was mounted onto a gelatin-coated glass slide and Nissl-stained with 0.1% cresyl violet. Muscimol injection sites were estimated based on the location of coagulation in coronal sections of the brains.

## Results

### Precise control of torque output is demanding

The behavioral task procedure is shown in Figure 1A. The correct rates for targets requiring precise torque control were significantly lower than other targets in both monkeys (Figure 1B) [(Monk-1:one-way ANOVA, *F*_4, 15_ = 17.94, *P* < 0.001; *Post hoc* multiple comparison: Moderate vs. More-Precise, *P* < 0.05; Moderate vs. High-Precise, *P* < 0.001; Precise vs. More-Precise, *P* < 0.05; Precise vs. High-Precise, *P* < 0.001; High vs. High-Precise, *P* < 0.001), (Monk-2: paired *t*-test, *t*_(8)_ = 6.45, *P* < 0.001)]. These results indicate that the precise control of torque output is demanding in both monkeys.

### Effect of the VSt inactivation on motor performance and cortical activity

To investigate whether the VSt has an impact on motor control, we inactivated the contralateral VSt to the task-engaged hand pharmacologically and compared performance and cortical activity between the control and the VSt inactivation sessions (Figures 2A,B).

Figure 2C shows a typical example of task-related modulations of the wrist torque (1st line panels: control, black; VSt inactivation, blue; data obtained from Monk-1) and oscillatory activity obtained from the M1 and PM sites (enlarged red and cyan circles in Figure 2A, respectively) in correct trials. Compared with control trials, torque outputs, except for the High-Precise target, were significantly decreased during the VSt inactivation trials (2nd line panels in Figure 2C). Torque outputs in the High-Precise target showed a similar trend, but the differences were not significant. In control trials, representative M1 and PM sites revealed increased activity in high-γ, γ, and θ bands around the go cue for all targets compared with baseline (rest period) activity (3rd and 6th line panels in Figure 2C). On the other hand, such modulation in high-γ and γ bands during VSt inactivation was decreased (4th and 7th line panels in Figure 2C). Statistical analysis showed a significant reduction of modulation in high-γ and γ bands during VSt inactivation compared to the control trials in both M1 and PM among targets (5th and 8th panels in Figure 2C). In contrast, significantly increased modulation in lower frequency bands, α and θ bands during the VSt inactivation trials, was observed in the M1 site only for Moderate, Precise, and More-Precise targets. A similar effect of VSt inactivation on both torque output and cortical activities was observed for the Moderate target in Monk-2. Torque outputs for the Precise target also showed a decreasing trend as well, but significant differences were not found in cortical activity (Supplementary Figure 1).

**Figure 3:**
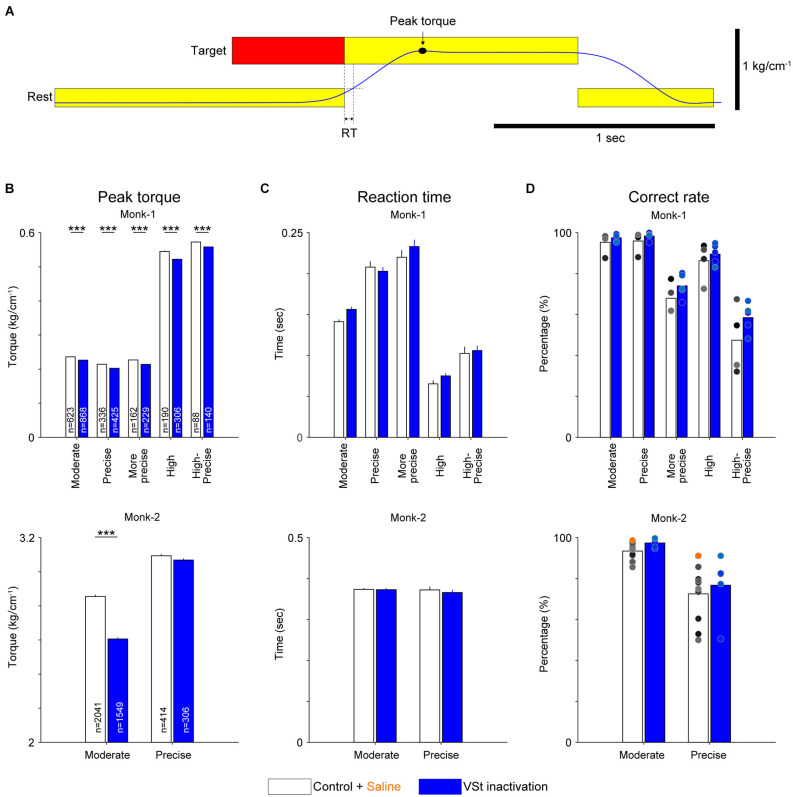
Population data of the effect of VSt inactivation on motor performances. **(A)** Definition of peak torque and reaction time (RT). Torque output is shown in blue. Peak torque is a maximum value of torque output revealed by a filled circle and arrow. RT is defined as the time window between the timing of the go cue and the timing when the cursor, which is associated with torque output, exceeded the rest position. **(B)** Peak wrist torque for each target in both monkeys (upper, Monk-1; lower, Monk-2). ‘n’ in each bar graph indicates the number of trials pooled across sessions. **(C)** RT for each target in both monkeys (upper, Monk-1; lower, Monk-2). In **(B)** and **(C)**, bar graphs and error bars indicate the mean and standard error of the mean, respectively. Wilcoxon-signed rank test (control vs. VSt inactivation) with Bonferroni correction was conducted for each target pair. ***P < 0.001. **(D)** Correct rate for each target in both monkeys (upper, Monk-1; lower, Monk-2). Individual dots indicate the results of each session (Monk-1: four Control sessions, five VSt inactivation sessions; Monk-2: nine Control sessions, one saline session, five VSt inactivation sessions).

Torque output traces in a typical example show a decreasing trend during VSt inactivation trials (1st and 2nd line panels in Figure 2C). We compared peak torque values (Figure 3A) of all trials for each target between control and inactivation sessions. The result in both monkeys showed significantly decreased peak torque for almost all targets (Figure 3B, Wilcoxon-signed rank test with Bonferroni correction; *P* < 0.001). Although peak torque values in correct trials for the Precise target in Monk-2 did not show significant change, they tended to be decreased compared with the control trials. To succeed in the trials for the Precise target by isometric contraction, the precise torque control, a minimal fluctuation of torque production was required. Therefore, a significant decrease in both the peak torque and high-frequency component in movement-related activity (Supplementary Figure 1) would not be observed in correct trials. Instead, the number of failed trials where the monkey could not maintain torque for the required time increased for the Precise target (Control, 7.3%; VSt inactivation, 11.8%), but not for the Moderate target (Control, 2.1%; VSt inactivation, 1.9%). These results suggest that the VSt inactivation interferes in maintaining torque output requiring precise control by isometric contraction.

In addition to the peak torque, VSt inactivation might affect the initiation of motor response, which is considered an indicator of motivational state (Hollerman et al., 1998; Hassani et al., 2001; Adcock et al., 2006; Mir et al., 2011). Thus, we analyzed the RTs (Figure 3A). VSt inactivation did not affect the RTs in both monkeys (Figure 3C, Wilcoxon-signed rank test with Bonferroni correction).

The correct rates for each target also showed no significant difference between the control and VSt inactivation sessions in both monkeys (Figure 3D, Wilcoxon-signed rank test with Bonferroni correction). This result seems consistent with previous findings showing that a VSt lesion or depletion of dopamine in the VSt did not affect instrumental responses requiring movements (Taylor and Robbins, 1986; Parkinson et al., 1999; Corbit et al., 2001; Cardinal and Cheung, 2005; Corbit and Balleine, 2011). Furthermore, omission trials were not increased by VSt inactivation [(Monk-1: Control, 1.38 ± 1.38% per session; VSt inactivation, 0.059 ± 0.059%), (Monk-2: Control, 4.26 ± 0.93%; VSt inactivation, 0%); average and standard error of the mean are shown]. These results combined with the results in RTs suggest that VSt inactivation does not induce a decrease in motivation to perform the task.

**Figure 4:**
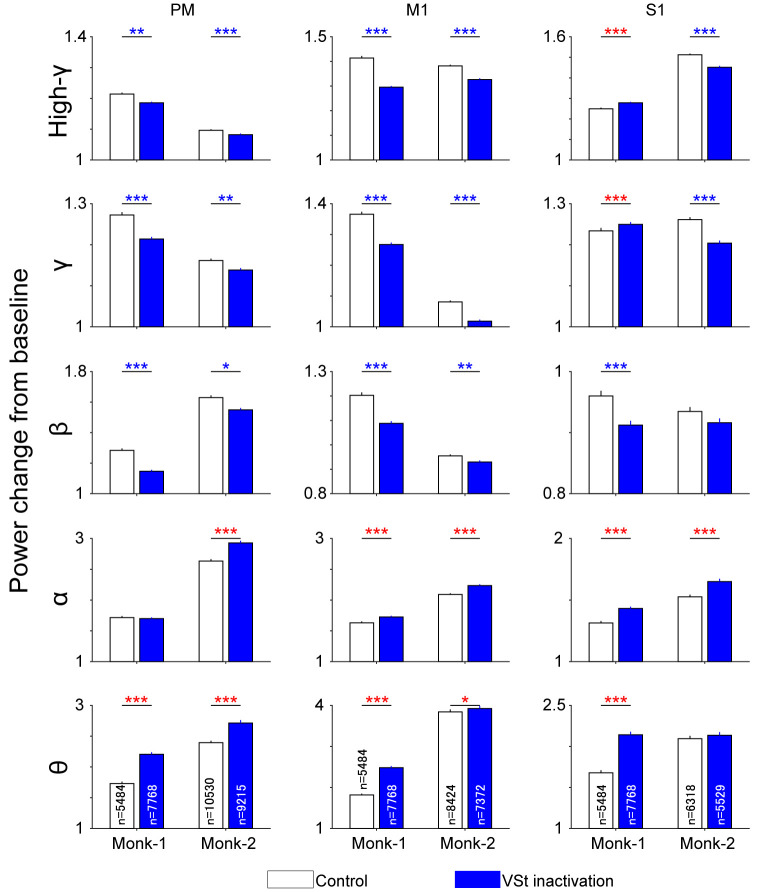
Population data of movement-related power change in the SMC. Power change around the go cue (Monk-1, −0.5 s to 0.5 s; Monk-2, 0 s to 1 s) from baseline were compared between control and VSt inactivation trials in each monkey. The frequency of interest was classified into the following five frequency bands, θ (5–7 Hz), α (8–14 Hz), β (15–30 Hz), γ (31–70 Hz), and high-γ (71–120 Hz). ‘n’ in bar graph indicates the number of trials × the number of channels in each area. Trials were pooled across sessions and targets and recording areas. Data are shown by the mean and standard error of the mean. Wilcoxon-signed rank test (control vs. VSt inactivation) was conducted for each panel. Blue asterisks indicate a significant decrease in VSt inactivation trials while red ones indicate an increase. *P < 0.05, **P < 0.01, ***P < 0.001. *P*-values are displayed in Supplementary Table 2.

Since the movement-related power change in representative sites in the M1 and PM showed a common reduction of high-frequency modulation among targets (Figure 2C), we combined the data for individual targets into a single set of data and compared the population data in the time window in which the movement-related power change was observed (−0.5 to 0.5 s and 0–1 s from the go cue in Monk-1 and Monk-2, respectively). The results showed similar changes in the PM and M1. VSt inactivation decreased movement-related power changes at high-γ, γ, and β bands (Figure 4, Wilcoxon-signed rank test, *P* < 0.01; *P*-values are shown in Supplementary Table 2). In contrast, the θ band in both the PM and M1 increased power changes during VSt inactivation in the two monkeys. While the S1 also showed similar changes in β, α, and θ bands, as well as the PM and M1, the difference in high-γ and γ bands between the VSt inactivation and control trials was contradictory in the two monkeys.

VSt inactivation may affect cortical activity in general during the rest phase of the task. Therefore, frequency power during the rest periods was analyzed. Frequency power at the β band in VSt inactivation trials was significantly decreased during the rest periods compared with control trials (Supplementary Figure 2; Wilcoxon-signed rank test, *P* < 0.001; *P*-values are shown in Supplementary Table 3), which was common in both monkeys except for Monk-2’s PM. In addition, high-γ power in the S1 increased in the VSt inactivation trials (Wilcoxon-signed rank test: Monk-1, *P* < 0.001; Monk-2, *P* < 0.01) while γ band power in the S1 decreased in both monkeys (Wilcoxon-signed rank test, *P* < 0.001). On the other hand, other frequency bands were contradictory in the two monkeys, although significant changes were instigated by VSt inactivation. This result indicates that SMC activities in the rest phase were also affected by VSt inactivation.

## Discussion

We investigated the functional role of the VSt in motor control by recording brain activity and motor performance. The present study demonstrated that, regardless of task difficulty, VSt inactivation reduces high-frequency movement-related oscillatory activities, which is accompanied by a decrease in torque output. Overall, our results provide direct evidence that the VSt regulates activities of the SMC and motor output.

Previous studies reported that manipulation of VSt activity did not affect general movements, such as feeding, locomotion, and lever pressing (Taylor and Robbins, 1986; Parkinson et al., 1999; Corbit et al., 2001; Cardinal and Cheung, 2005; Corbit and Balleine, 2011). Indeed, as in previous studies, VSt inactivation did not affect the correct rate (Figure 3D). However, VSt inactivation decreased torque output and high-frequency activity of motor cortices and increased low-frequency activity (Figures 2C, 3B, and 4), suggesting that the VSt might have motor function as a source of motor outputs *via* the motor-related areas. While the mechanism by which increased low-frequency activity associates with decreased torque output by VSt inactivation is still unclear, the high-frequency component of the ECoG signal reflects synchronized firing of a neural population near the electrode (Leuthardt et al., 2004; Ray et al., 2008) and represents muscle activity (Shin et al., 2012). Our finding that a reduction of high-frequency and an enhancement of low-frequency components of motor cortices occurred in association with decreased torque output suggests that the VSt plays an important role in regulating the extent of torque output.

The pathway from the VSt to the SMC, especially to the M1, is unclear. Anatomical and electrophysiological studies may provide potential pathways. The macaque VSt has minimally disynaptic projection to the M1, presumably *via* basal forebrain cholinergic neurons (Miyachi et al., 2005). In addition, the rodent VSt increases or decreases the M1 neuronal firing rate through the VSt-medial substantia nigra reticulata-motor thalamus-M1 pathway (Aoki et al., 2019). An additional potential pathway is the neuronal pathway that relays the ventral midbrain. The VSt and the ventral midbrain containing abundant dopamine neurons have mutual connectivity (Lynd-Balta and Haber, 1994a, b, c; Haber and Knutson, 2010). Furthermore, the ventral midbrain has disynaptic projection to spinal neurons *via* the M1 (Suzuki et al., 2022), suggesting that the VSt may impact the M1 and motor control through the ventral midbrain. These pathways might account for the relationship between the VSt and descending commands.

Perturbation of the nucleus accumbens, a part of the VSt, disrupts the exertion of effort in rodents measured by fixed-ratio lever press schedules (Aberman and Salamone, 1999; Cardinal et al., 2001; Salamone et al., 2001; Ishiwari et al., 2004) and climbing a barrier to obtain reward (Hauber and Sommer, 2009). In the task of the present study, precise torque control to sustain torque production with small variation is a highly demanding motor control requiring greater effort. Here, we hypothesized that a demanding task, such as showing lower correct rates in control sessions (Figure 1B), would be affected by VSt inactivation. However, as mentioned above, VSt inactivation did not affect the correct performance rate in all targets (Figure 3D). Rather, regardless of task difficulty, VSt inactivation reduced torque output (Figures 2C, 3B), which may reflect the exertion of less physical effort as reported in previous studies. This interpretation can be helpful for understanding the behavior of depressive patients. Malfunction of the VSt is reported in patients with depression (Epstein et al., 2006; Wacker et al., 2009; Hwang et al., 2016). Depressed patients are not able to produce the same level of physical effort measured by grip force as healthy subjects for larger rewards. Since they behave as if they do not want to gain large rewards, it is suggested that incentive motivation impairment is a core deficit of depression (Cléry-Melin et al., 2011). However, as revealed by the present study, less physical effort exertion in depressed patients might also reflect evidence that the VSt contributes to movement related activity of the motor cortices. Furthermore, it is reported that the VSt is required for controlling recovered precision grip after spinal cord injury in monkeys. In the recovery stage, a higher level of VSt and M1 activity for the execution of the same movement before injury is required (Nishimura et al., 2007; Sawada et al., 2015; Suzuki et al., 2020). In the light of the present finding, both in the intact state and after injury, the VSt likely serves as a regulator of motor cortex activity to achieve a motor task.

The RTs, frequently used as an indicator of motivation (Hollerman et al., 1998; Hassani et al., 2001; Adcock et al., 2006; Mir et al., 2011), were not affected by VSt inactivation in the present study. Furthermore, VSt inactivation did not increase omission trials. These results indicate that motivation is not altered by VSt inactivation as demonstrated in previous studies (Taylor and Robbins, 1986; Parkinson et al., 1999; Corbit et al., 2001; Cardinal and Cheung, 2005; Corbit and Balleine, 2011). Therefore, the function of the VSt might be as a modulator of motor circuits and, then, effort-related behaviors rather than as a simple motivational node.

VSt inactivation also decreased β band activity in the SMC during the rest period, although the mechanism of this effect is unclear. VSt inactivation might affect the preparatory activity for movements, causing reduced torque output.

In conclusion, we have uncovered a new aspect of VSt function in regulating SMC activities and torque output. The present study provides new data to understand the mechanism of generating motivated behavior, motivation-driven effort, and psychiatric disorders accompanied by motor symptoms.

## Data Availability Statement

The raw data supporting the conclusions of this article will be made available by the authors, without undue reservation.

## Ethics Statement

The animal study was reviewed and approved by the Committees for Animal Experiments at the National Institute of Natural Sciences and at the Graduate School of Medicine in Kyoto University.

## Author Contributions

MS designed and conducted the experiments, performed the surgeries, analyzed and interpreted the data, and wrote the manuscript. YN designed the experiments, performed the surgeries, interpreted the data, and wrote the manuscript. All authors contributed to the article and approved the submitted version.

## Funding

This work was supported by grants from Japan Society for the Promotion of Science (JSPS) KAKENHI (Grant number: 20H05714, 20H05489, 18K19767, 18H05151 to YN and 17J05341 and 19K18410 to MS).
