# Supplementary material for: The ventral striatum contributes to the activity of the motor cortex and motor outputs in monkeys
Source: Front Syst Neurosci. 2022 Aug 29;16:979272. doi: 10.3389/fnsys.2022.979272 (PMC9540202; doi:10.3389/fnsys.2022.979272)
Supplement: Supplementary file 1 [file Data_Sheet_1.docx]

Supplementary Material

# Supplementary Figures and Tables

## Supplementary Figures


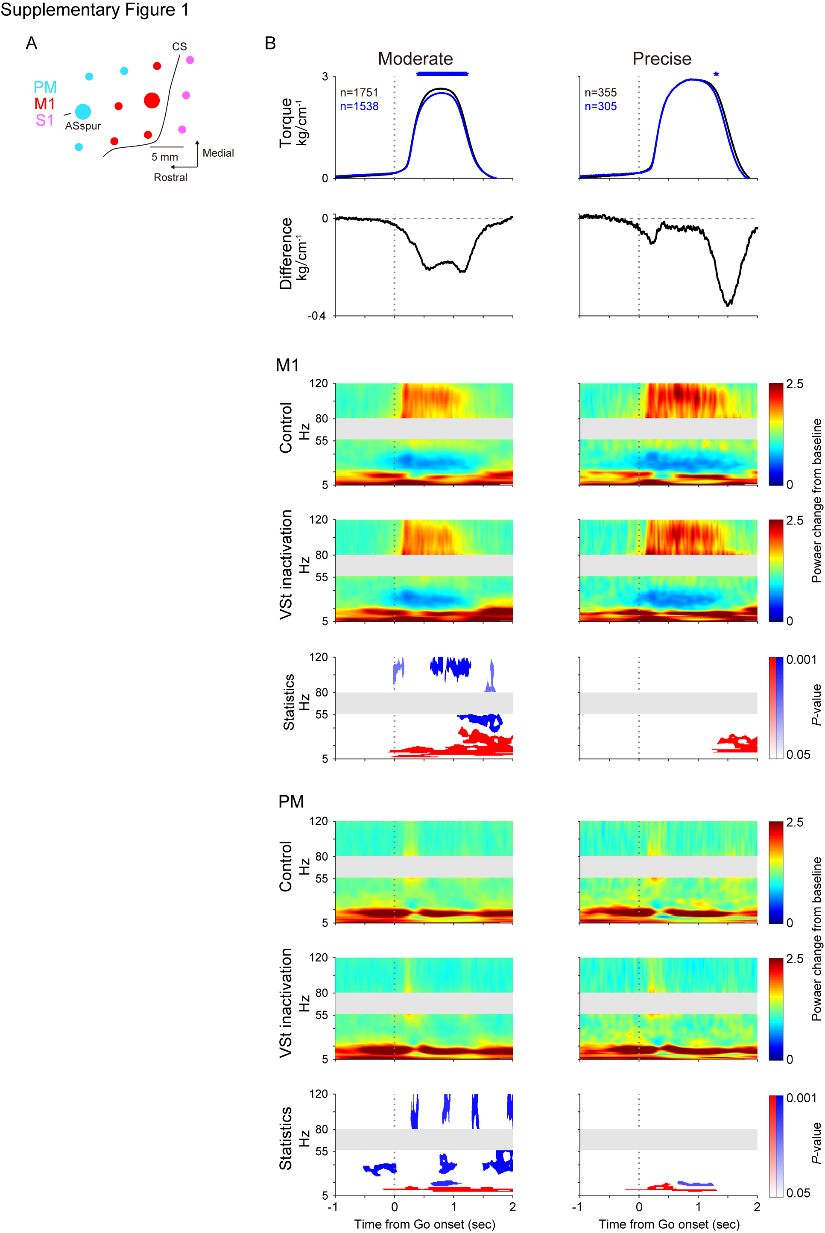


**Supplementary Figure 1.** **VSt inactivation reduces torque output and movement-related activity (Monk-2).** (**A**) Recording sites on the PM, M1, and S1 are shown in cyan, red, and pink color, respectively. The electrode locations on the functional map were determined relative to anatomical landmarks such as the central sulcus and arcuate sulcus and the location of the electrode. The activity of a typical example in (B) were recorded from the enlarged red (M1) and cyan (PM) sites. CS, central sulcus; ASspur, spur of the arcuate sulcus. (**B**) Comparison of movement-related activity of a typical example in the M1 and PM between control and the VSt inactivation trials. First row: Averaged wrist torque trace (Control, black; VSt inactivation, blue). Statistical difference between control and inactivation was investigated by Wilcoxon-signed rank test for wrist torque values from 0 sec before to 1.5 sec after go cue (376 time points) were conducted with Bonferroni correction (Control vs VSt inactivation). Blue asterisks indicate significant reduction in torque output during the VSt inactivation (*P* < 0.001). There were no time points with higher torque output during VSt inactivation compared with the control trials. Second row: Difference of torque traces between the inactivation and control (VSt inactivation minus Control). Negative value indicates reduced torque output during the VSt inactivation. Third, fourth, sixth and seventh rows: Averaged movement-related power change of oscillatory cortical activity during the control (M1, third; PM, sixth row) and VSt inactivation (M1, fourth; PM, seventh row) trials. Each time-frequency representation value was divided by the mean baseline (rest period: -1.9 s to -1.3 s before go cue) value, then averaged across trials (see also ECoG analysis in Materials and Methods). Fifth and eighth rows: Statistical *P*-value representation in time-frequency domain (M1, fifth; PM, eighth). Data at 55-80 Hz in Monk-2 were excluded due to extraordinal signal contamination. Blue and red areas indicate statistically inactivated and activated components during the VSt inactivation, respectively. Trials showing abnormal spectra were excluded using an automated algorithm from the EEGLAB library, pooled across sessions, and analyzed.


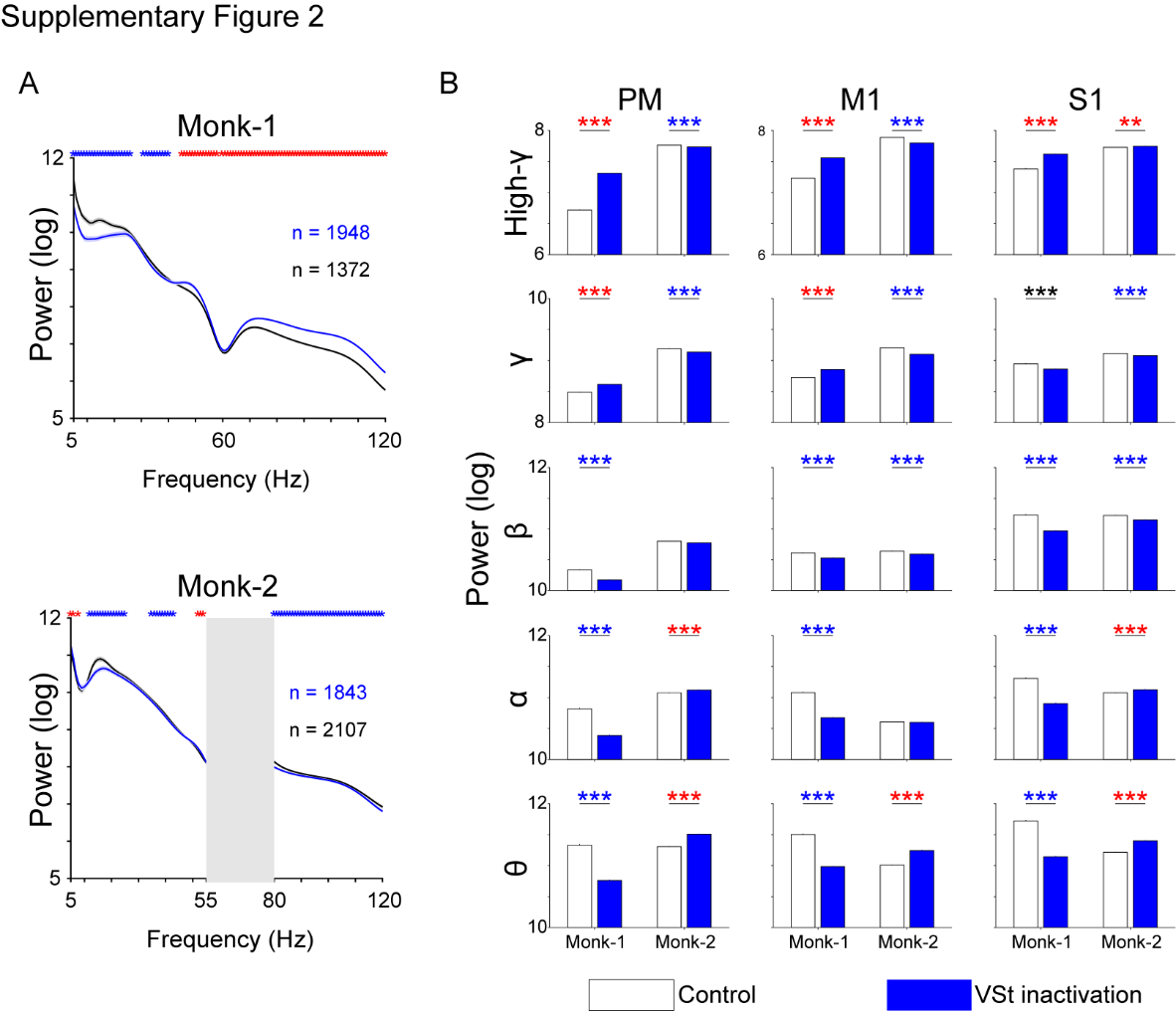


**Supplementary Figure 2.** **Population data of effect of VSt inactivation on activities of the SMC during rest phase.** (**A**) Example of power for each frequency during the rest period of the task. Data obtained from a representative recording site in the M1 shown in Figure 2A (Monk-1) and Supplementary Figure 1A (Monk-2). Data at 55-80 Hz in Monk-2 were excluded due to extraordinal signal contamination. ‘n’ in each panel indicates the number of trials. Wilcoxon-signed rank test with Bonferroni correction was conducted at each frequency. Blue asterisks indicate significant decrease of power in VSt inactivation trials while red ones indicate increase (*P* < 0.05). (**B**) Population data of power for each frequency band in individual areas. Data are shown by mean and standard error of the mean. Legends were the same with Figure 4. **; *P* < 0.01, ***; *P* < 0.001. *P*-values are displayed in Supplementary Table 3.

## Supplementary Tables

**Supplementary Table 1. Number of trials in each session and target information**

| Monkey 1  Session | Number of trials (Correct trials) | Target size (mm × mm),  Distance from rest position (mm, center to center) |
| --- | --- | --- |
| Control 1 | 451 (404) | Moderate; 156 × 44, 130  Precise; 44 × 44, 72  More precise; 23 × 44, 72  High; 62 × 44, 180  High-Precise; 23 × 44, 180 |
| Control 2 | 305(241) |  |
| Control 3 | 408 (370) |  |
| Control 4 | 481 (384) |  |
| Inactivation 1 | 419 (366) |  |
| Inactivation 2 | 457 (405) |  |
| Inactivation 3 | 460 (412) |  |
| Inactivation 4 | 441 (407) |  |
| Inactivation 5 | 437 (378) |  |
|  | | |
| Monkey 2  Session | Number of trials (Correct trials) | Target size (mm × mm),  Distance from rest position (mm, center to center) |
| Control 1 | 361 (333) | Moderate; 234 × 44, 189  Precise; 44 × 44, 112 |
| Control 2 | 231 (175) |  |
| Control 3 | 255 (219) |  |
| Control 4 | 318 (296) |  |
| Control 5 | 285 (256) |  |
| Control 6 | 306 (292) |  |
| Control 7 | 190 (164) |  |
| Control 8 | 159 (124) |  |
| Control 9 | 258 (234) |  |
| Saline | 373 (362) |  |
| Inactivation 1 | 390 (373) |  |
| Inactivation 2 | 402 (339) |  |
| Inactivation 3 | 395 (368) |  |
| Inactivation 4 | 400 (379) |  |
| Inactivation 5 | 394 (387) |  |

**Supplementary Table 2. Statistical P-values in movement-related power change**

| Wilcoxon-signed rank test | PM | | M1 | | S1 | |
| --- | --- | --- | --- | --- | --- | --- |
|  | Monk-1 | Monk-2 | Monk-1 | Monk-2 | Monk-1 | Monk-2 |
| High-γ | *P* = 1.67 × 10^-3^ | *P* = 9.76 × 10^-4^ | *P* = 7.69 × 10^-14^ | *P* = 1.37 × 10^-14^ | *P* = 5.88 × 10^-4^ | *P* = 1.92 × 10^-16^ |
| γ | *P* = 1.14 × 10^-7^ | *P* = 2.01 × 10^-3^ | *P* = 3.39 × 10^-17^ | *P* = 4.97 × 10^-25^ | *P* = 5.79 × 10^-4^ | *P* = 5.52 × 10^-13^ |
| β | *P* = 3.66 × 10^-21^ | *P* = 0.0256 | *P* = 8.94 × 10^-31^ | *P* = 1.87 × 10^-3^ | *P* = 3.52 × 10^-10^ | *P* = 0.49 |
| α | *P* = 0.89 | *P* = 2.06 × 10^-30^ | *P* = 5.46 × 10^-7^ | *P* = 2.25 × 10^-8^ | *P* = 1.37 × 10^-7^ | *P* = 1.67 × 10^-4^ |
| θ | *P* = 3.00 × 10^-49^ | *P* = 3.46 × 10^-11^ | *P* = 1.00 × 10^-66^ | *P* = 0.0468 | *P* = 6.87 × 10^-42^ | *P* = 9.12 × 10^-64^ |

**Supplementary Table 3. Statistical P-values in frequency power during rest period**

| Wilcoxon-signed rank test | PM | | M1 | | S1 | |
| --- | --- | --- | --- | --- | --- | --- |
|  | Monk-1 | Monk-2 | Monk-1 | Monk-2 | Monk-1 | Monk-2 |
| High-γ | *P* < 10^-200^ | *P* = 3.19 × 10^-8^ | *P* < 10^-200^ | *P* = 3.59 × 10^-55^ | *P* = 4.97 × 10^-30^ | *P* = 1.68 × 10^-3^ |
| γ | *P* = 5.41 × 10^-47^ | *P* = 3.57 × 10^-14^ | *P* = 1.75 × 10^-26^ | *P* = 1.98 × 10^-54^ | *P* = 4.33 × 10^-24^ | *P* = 1.86 × 10^-6^ |
| β | *P* = 1.04 × 10^-50^ | *P* = 0.22 | *P* = 1.04 × 10^-20^ | *P* = 1.18 × 10^-9^ | *P* = 1.42 × 10^-44^ | *P* = 8.13 × 10^-8^ |
| α | *P* = 2.18× 10^-162^ | *P* = 9.13 × 10^-5^ | *P* = 3.83 × 10^-171^ | *P* = 0.51 | *P* = 1.27 × 10^-114^ | *P* = 4.90 × 10^-5^ |
| θ | *P* = 7.69 × 10^-212^ | *P* = 2.41 × 10^-56^ | *P* = 6.85 × 10^-195^ | *P* = 2.77 × 10^-76^ | *P* = 4.93 × 10^-191^ | *P* = 9.12 × 10^-64^ |
